# Effect of Different Ultrasonic Power on the Properties of RHA Steel Welded Joints

**DOI:** 10.3390/ma15030768

**Published:** 2022-01-20

**Authors:** Peng Yin, Chunguang Xu, Qinxue Pan, Wenjun Zhang, Xiaowei Jiang

**Affiliations:** 1 Key Laboratory of Fundamental Science for Advanced Machining, Beijing Institute of Technology, Beijing 100081, China; yin_peng1991@163.com (P.Y.); xucgbit@263.net (C.X.); zwj787404079@163.com (W.Z.); jiangxiaowei_bit@126.com (X.J.); 2 Jiangsu Institute of Automation, Lianyungang 222061, China

**Keywords:** ultrasonic wave, homogeneous armor steel, power, microstructure, mechanical properties

## Abstract

Based on the changes of microhardness, tensile strength, and impact resistance caused by the difference of macroscopic morphology and microstructure of welded joints, this paper studied the effect of different ultrasonic power on the properties of welded joints during the welding of homogeneous armor steel. It is experimentally found that the macroscopic morphology of those joints is very different. Compared with conventional welding, ultrasonic welding can increase the weld depth and the width of the heat-affected zone (HAZ) on either side of the weld. However, only the ultrasonic wave at an appropriate power level can increase the weld width. In addition, appropriate ultrasonic power can significantly improve the grain state of the weld. With the increase of ultrasonic power, the grain size in HAZ will decrease. The microhardness of the weld will first increase and then decrease, while the microhardness of the HAZ will increase. This is basically consistent with the changing trend of impact resistance. An ultrasonic wave can also increase the tensile strength of a welded joint up to 802 MPa, 12.4% higher than that in conventional welding. However, a high-power ultrasonic wave will bring down the tensile strength. This study provides guidance for the selection of ultrasonic-assisted regulation power to achieve the different properties of homogeneous armor steel joints.

## 1. Introduction

Since the formal development of armor protection technology in the late 19th century, rolled homogeneous armor (RHA) steel has been widely used in the main components of tanks and armored vehicles due to its excellent mechanical properties, such as high hardness and high strength. Weldability has become the main performance index of this kind of steel [1]. For different parts of the same equipment, the requirements of welding performance are different. For example, high hardness and wear resistance is required for some parts, while high strength and impact resistance or good comprehensive performance is required for the other parts. Therefore, how to quickly and conveniently produce the RHA steel welds with specific welding performance at low cost and develop an efficient welding process to solve the above problems has always been a hot spot in the field of weapon research in various countries.

However, the RHA steel has high carbon equivalent and poor weldability, so the RHA steel welds are prone to cracking and incomplete penetration [2]. Moreover, due to the influence of welding heat transfer, the grain state of each region of a welded joint is poor. These problems seriously affect the welding performance and reduce the service reliability of weapons, thus becoming the difficulties in the research of RHA welding performance. At present, the research on RHA welding performance control is mainly focused on the optimization of process parameters and fixtures [3,4,5,6,7]. However, different welding conditions and fixtures have different effects on the welding performance, so a good unified control method is currently unavailable.

In recent years, scholars have carried out a lot of research on low-frequency vibration-assisted welding in order to obtain high welding performance. The results show that the microstructure and mechanical properties of welds cannot be improved, either by global vibration or local vibration. However, in some specific cases, the small deformation caused by vibration can reduce the welding stress [8]. With the change of vibration mode and frequency, low-frequency vibration welding can improve the toughness of welded joints without negative impact on other mechanical properties [9]. It has also been reported that applying an electromagnetic field in the welding process can effectively refine the weld grains and improve the weld hardness but, unfortunately, will weaken some mechanical properties of the weld, including strength and toughness [10,11]. All the above vibration is applied to the base metal and acts on the weld area through the base metal, but the effect of weld regulation is limited and cannot meet the requirements of RHA welding performance.

In addition to the above studies, many scholars also applied vibration directly to the arc to achieve the so-called laser oscillation welding, which produced good welding effects [12,13]. The different properties of a weld can be improved by using the laser beams in different oscillation modes to stir the weld pool. Transverse laser oscillation welding can refine the grain size of columnar crystals and slightly improve the microhardness and strength of welded joints [14]. Annular laser oscillation can effectively reduce the peak welding temperature and the temperature gradient in weld pool cooling [15]. However, the influence of different vibration modes on the microstructure and properties of welded joints has not been further studied, especially for RHA steel materials.

It was found with the development of ultrasonics that ultrasonic casting could effectively improve the microstructure and properties of metals [16,17,18]. Subsequently, scholars began a lot of research on ultrasound-assisted welding. According to most of the reports, the welds could be ultrasonically regulated in two ways. One method was to apply ultrasonic waves to the arc, so as to change the arc shape in the welding process and obtain higher melting energy [19,20,21,22,23]. Subsequently, it was found that the weld width obtained by such method was increased, and that the weld microstructure was significantly refined, due to ultrasonic effect and gradually became coarsened with the increase of ultrasonic frequency [24]. With the increase of ultrasonic power, the weld porosity would first decrease and then increase, but the weld strength would continue to increase [25]. According to the analysis, the above results were mainly caused by the fact that the morphology and distribution of materials were affected by cavitation and ultrasonic flow during ultrasonic arc welding [26]. Another method was to apply ultrasonic waves to the interior of the weld pool to improve the weld-cooling rate, refine the weld microstructure, and greatly improve the material uniformity, thus changing the grain size and hardness of the weld and HAZ and improving the tensile strength [27,28,29,30,31]. Meanwhile, ultrasonic vibration would not change the phase composition of the weld zone but would affect the grain growth orientation and phase content [32,33]. Jian et al. suggested that not only the cavitation effect but also the heteronucleation induced by ultrasonic cavitation was responsible for grain refinement [34]. Kumar pointed out that the current research on ultrasound-assisted welding was mostly limited to soft metals, such as aluminum, and that the application of ultrasonic process to the individual variants of harder and more complex materials was still in the early exploration stage [35]. The research on applying ultrasonic vibration to RHA steel welding has not been found yet.

However, none of the above studies can play a guiding role in regulating the welding performance of homogeneous armor steel with high strength and hardness. In order to obtain the welding joints with different welding properties and high welding efficiency, an ultrasonic wave was applied in this study to the base metal under welding to regulate the welding performance. The influence of ultrasonic power on the macroscopic morphology, microstructure, and mechanical properties of RHA welded joints was systematically studied, and the causes for different welding performance were analyzed. This study provides guidance for the selection of ultrasonic power to achieve the different properties of homogeneous armor steel joints.

## 2. Experimental Procedures

### 2.1. Materials

**Figure 1:**
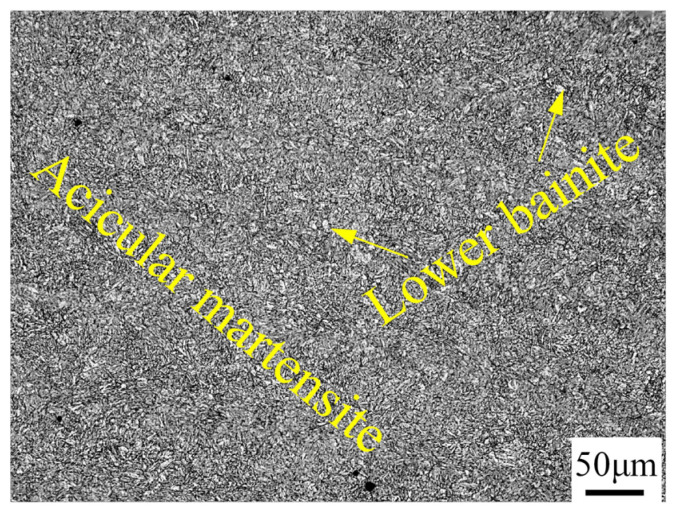
Microstructure of base mental.

The base mental (BM) used in this test is a 30CrNiMoNb homogeneous armor plate that has been quenched and tempered at low temperature. According to GJB 8486-2015, its brand number is defined as 6211. The microstructure of 6211, as shown in Figure 1, is mainly composed of acicular martensite and a small amount of granular bainite. The size of the base material is 310 mm × 15 mm × 5 mm, and the butt end faces have a 30° Y-shaped groove (or single V groove with broad root face) with a 1-mm root face. The welding wire material is HCr20Ni10Mn7Mo high-hardness austenitic stainless steel wire with a diameter of 1.2 mm. The main chemical composition of base material and welding wire is shown in Table 1.

### 2.2. Test System

**Figure 2:**
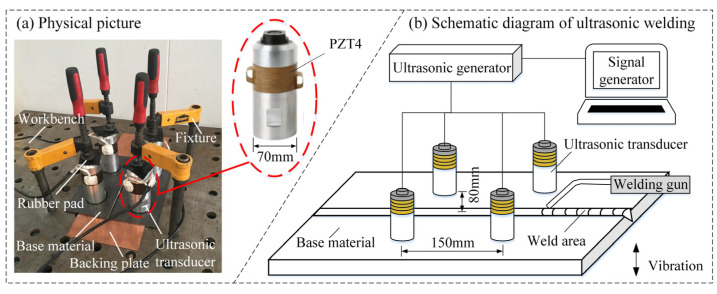
Physical configuration and overall sketch of test system.

The test system consists of a welding system and an ultrasound-assisted system. The welding system is a PANA-AUTO KRⅡ500 welding machine (MIG/MAG) produced by Panasonic (Osaka, Japan). The ultrasound-assisted system consists of a signal generator, an ultrasonic generator, and an ultrasonic transducer. The signal generator is a RIGOL DG4102 function/arbitrary waveform signal generator, which can output 1 μHz–60 MHz arbitrary waveform signals. For the ultrasonic generator, its maximum output power is 3000 W, and its output frequency range is (0–30) kHz. The ultrasonic transducer is a PZT4 piezoelectric ceramic transducer with a diameter of 70 mm and a resonant frequency of 14.85 kHz. The physical configuration before welding preparation is shown in Figure 2a, and the overall sketch of test system is shown in Figure 2b.

### 2.3. Test Method

**Figure 3:**
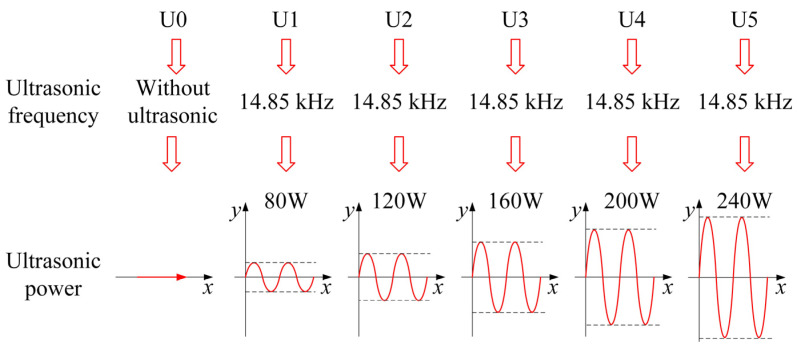
Parameters of the ultrasonic waves applied during welding.

Because the base metal is thin, single-pass welding is adopted in this study. Before welding, oxidation film and oil stains are removed from the surface of the base metal through mechanical grinding and acetone wiping. To prevent the welding damage to the worktable surface and ensure good weld formability, a flat and smooth copper backing is placed under the base metal with a butt clearance of 1 mm. The ultrasonic transducers with a buttered bottom are distributed on the front side of the base material, as shown in Figure 2b. An insulating fixture is used to fix each transducer until the transducer bottom is in close contact with the base metal surface. Then, the ultrasound-assisted system is turned on for welding according to the welding parameters shown in Table 2. To study the effect of different ultrasonic power on the macroscopic morphology, microstructure, and mechanical properties of RHA welded joints in the welding process, six groups of experiments have been designed. The first group is a control experiment without ultrasonic application. In experiment groups 2–6, the ultrasonic waves with different power strengths are applied in the welding process. The specific group numbers and ultrasonic settings are shown in Figure 3.

**Figure 4:**
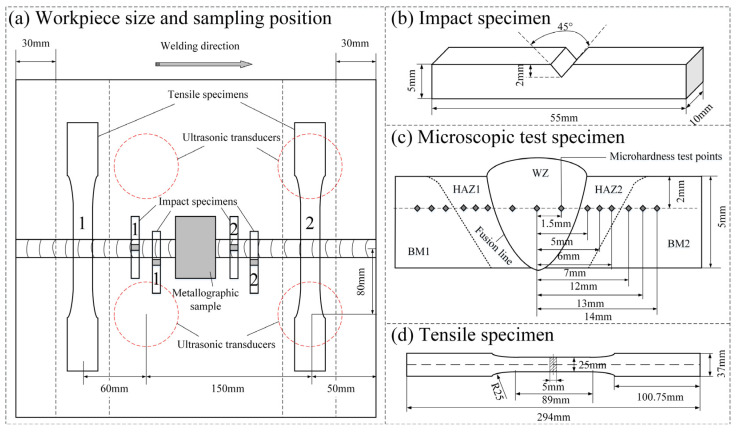
Sketch of experimental sample.

**Figure 5:**
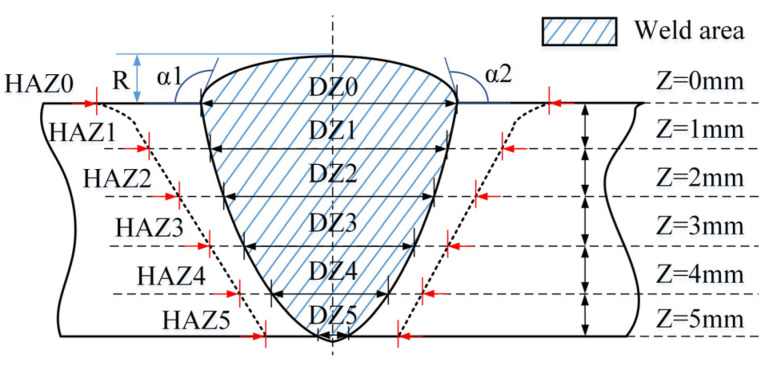
Positions of macroscopic deformation zones of a welded joint.

After the welding is completed, the test plates are sampled by line cutting at the specific sampling locations shown in Figure 4a. A total of 12 tensile samples, 12 WZ impact samples, 12 HAZ impact samples, and 6 metallographic samples are obtained from 6 groups of test plates. Figure 4b shows the Charpy pendulum impact specimens for weld zone (WZ) and heat-affected zone (HAZ), which shall be impacted by a SANS PTM2200-D1 Charpy tester at room temperature according to ISO 148-1:2016. After the section of the sample joint is polished, the sample is corroded by 4% nitric acid ethanol solution, observed by an OLYMPUS GX51 metallographic microscope, and then tested by a Vickers hardness tester according to ISO 6507-1:2005 to measure its microhardness. The test load is 10 kg, and the loading time is 15 s. The samples and microhardness test points are shown in Figure 4c. The tensile sample, whose size is shown in Figure 4d, is tested by a SANS SHT4305 machine at the test speed of 1 mm/min according to ISO 4136:2001. In addition, to better understand the fracture behavior, the fracture surface of the tensile sample is analyzed by a QUANTA FEG450 field emission scanning electron microscope (SEM), and the fracture surface elements are qualitatively and quantitatively analyzed at fast speed by EDAX’s energy dispersion spectrum (EDS) platform. Figure 5 shows the deformation positions at which the macroscopic features of the welded joint are measured, including the WZ width and HAZ width at different depths of the weld section, the left weld toe angle (α1) and right weld toe angle (α2), the heap height (R), and the shaded weld area.

## 3. Results and Discussion

### 3.1. Macroscopic Morphology of a Weld

**Figure 6:**
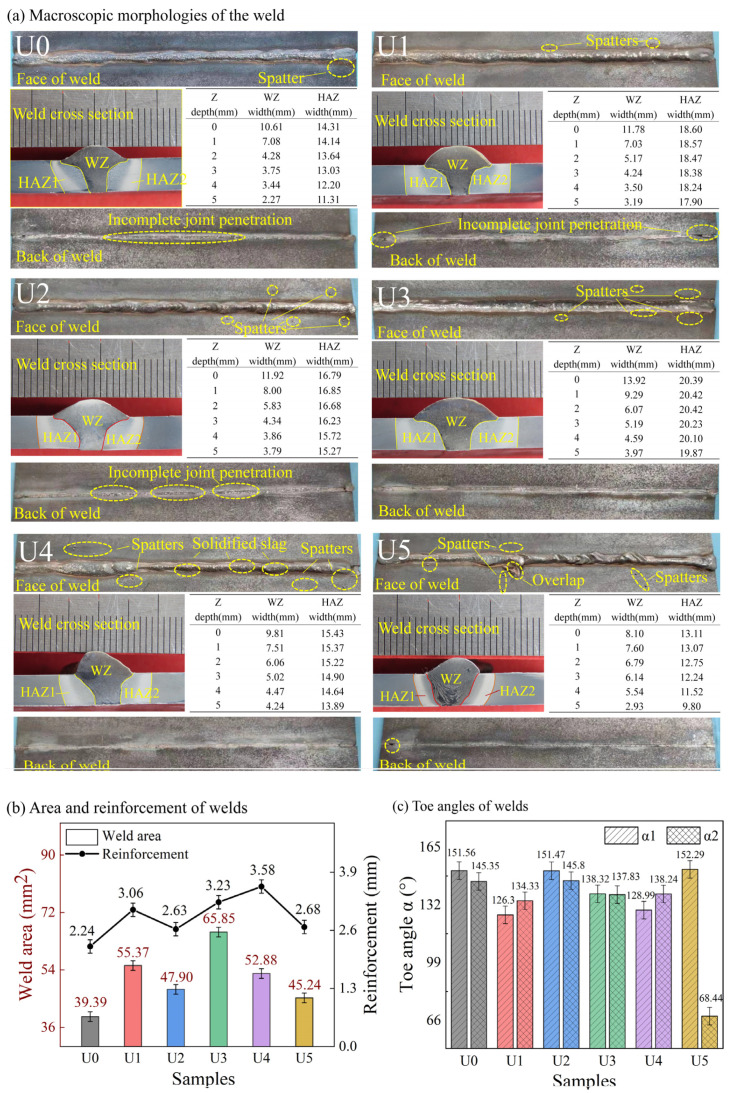
Macro-profile and size of a weld.

The macro-profiles of the welds formed by conventional welding and ultrasonic-assisted welding, as shown in Figure 6, are quite different in terms of the deformation size in each zone and the weld-forming quality. It can be seen from Figure 6a that the face of the U0 weld has a good profile without obvious defects in any area, except for the spatters at the arc-starting and arc-ending points, but the back of weld has a long incomplete joint penetration. The U1–U5 welds (from lower power to higher power) are ultrasonically regulated. With the increase of ultrasonic power, the spatter on weld face will become more and more serious. When the power increases to U4 and higher, the weld formability will be very poor. This is true especially when the ultrasonic power increases to U5, at which the melted metal will flow to the unmelted base metal outside the weld and form an overlap. It can be observed from the back of the weld that, when the ultrasonic power is low, the local incomplete joint penetration will occur, as shown in the U1 and U2 cases in Figure 6a. However, when the power increases to U3 and higher, the phenomenon of incomplete joint penetration will disappear.

As can be seen from the cross sections of the welded joints in Figure 6, the HAZs on both sides of the conventional weld are obviously asymmetric, but the HAZs on both sides of the ultrasonically regulated weld are very symmetric. This is mainly because the arc energy is evenly distributed to both sides of the weld by the acoustic flow effect caused by the transducers arranged symmetrically on both sides of the weld [36]. Except for the bowl-shaped U5 welds, the cross sections of all welds are in a regular funnel shape. As can be seen from the data in Figure 6, the widths of both WZ and HAZ will increase first and then decrease with the increase of ultrasonic power. U3 is the turning point of this trend. When Z = 0 mm and Z = 5 mm, the WZ width of the U3 weld will increase by 32% and 75%, and the HAZ width will increase by 43% and 60%, respectively, compared with the U0 weld. However, when Z = 0 mm, the WZ width of the U5 weld will decrease by 24%, and the HAZ width by 8%, compared with the U0 weld. When Z = 5 mm, the WZ width of the U5 weld will increase by 29% compared to the U0 weld. Therefore, it can be inferred that ultrasonic regulation can increase the weld width, especially when Z = 5 mm. This also indicates that the ultrasonic energy propagates unevenly in the weld pool. This phenomenon is related to the thickness of the base metal and the surface tension of the metal melt, as well as the ultrasonic-induced change in the driving force of the melt flow in the weld pool [23].

With the change of weld width, other morphologic parameters of a weld will also change. The analysis of data in Figure 6b,c shows that ultrasonic regulation can increase the weld area and reinforcement height. It has also been mentioned in previous studies that ultrasonic wave can increase the energy of weld pool diffusion [37]. However, with the increase of ultrasonic power, the weld area may not increase. In this study, the area of the U3 weld is the maximum. It is also found that the toe angles (α) of ultrasonically regulated welds are smaller than those of conventional welds. In particular, the toe angle of the U5 weld is almost an acute angle. This is because the surface tension of the melt is changed by cavitation effect and acoustic streaming effect during ultrasonic regulation, thus steepening the toe angle. As a result, the residual stress of the weld toe will undoubtedly increase to a certain extent, which is also very regrettable.

### 3.2. Weld Microstructure

**Figure 7:**
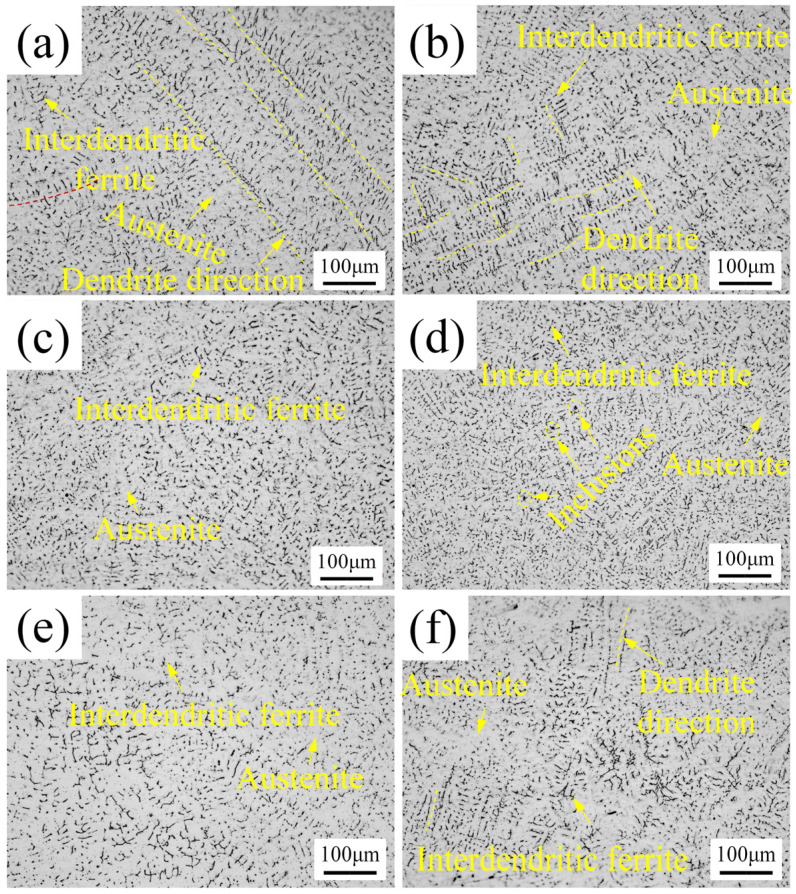
Weld microstructure: (**a**–**f**) U0–U5, respectively.

The microstructure of a weld zone of austenitic stainless steel is mainly composed of austenitic dendrite matrix and interdendritic ferrite. Interdendritic ferrite can clearly show the outline of austenite dendrite. The state of weld microstructure can be evaluated by judging the size and distribution of ferrite. Figure 7a–f, respectively, show the microstructures of the U0–U5 welds. Some grains of the U0 weld are relatively small and are evenly distributed, as shown in the lower left of Figure 7a. However, some grains are coarse, with very strong global directivity and incomplete fragmented original dendrites. The grain state of the U1 weld is similar to that of the U0 weld, as well as has some areas with strong grain directivity, which, however, are obviously improved compared with the U0 weld. The areas with strong grain directivity are obviously smaller, alternately distributed, and approximately vertical, as shown in Figure 7b. In the U2 weld, the grain distribution is much better and more uniform, but some grains are coarse. The microstructure of the U3 weld is shown in Figure 7d. It can be seen that the U3 weld is in the best condition and that its grains are fine and uniformly distributed. In the U4 weld, the interdendritic ferrite begins to aggregate, the grains grow up, and the grain distribution in some areas is poor. In the U5 weld, some interdendritic ferrite is clustered together, and the grains are coarse and unevenly distributed.

It can be seen that, due to the acoustic streaming effect and cavitation effect of ultrasonic wave [38], the ultrasonic wave propagating in the weld melt can regulate the weld microstructure, and the regulation effect is closely related to the power of ultrasonic wave. The ultrasonic wave with low power plays a small role in refining the grains and improving the grain distribution. With the increase of ultrasonic power, the weld grains become smaller and more uniformly distributed, and the regulation effect is better. However, the power increase has a threshold. Once the threshold is reached, the increase of ultrasonic power will result in a worse grain state featuring the growth and maldistribution of grains. As the power continues to increase, the grain state becomes worse and worse. This phenomenon is caused by the intensity of acoustic stream and cavitation. Theoretically, with the increase of ultrasonic power, the energy of ultrasonic flow and cavitation in the melt will increase [18]. The low ultrasonic power is not enough to produce very strong cavitation bubbles, so the effect of grain fragmentation is not good. Moreover, the energy of acoustic flow is not large enough to control the grain distribution. As the power of ultrasonic wave increases, the energy of ultrasonic wave will increase to fully satisfy the energy requirement of grain refinement. Moreover, the acoustic flow with high energy will take the refined grains back into the uncured melt and evenly distribute them. However, when the power exceeds the threshold, a high-intensity acoustic stream will be generated. The acoustic stream in this state will gather the grains together and push them to the direction of acoustic propagation quickly, resulting in rapid grain growth and poor grain distribution.

**Figure 8:**
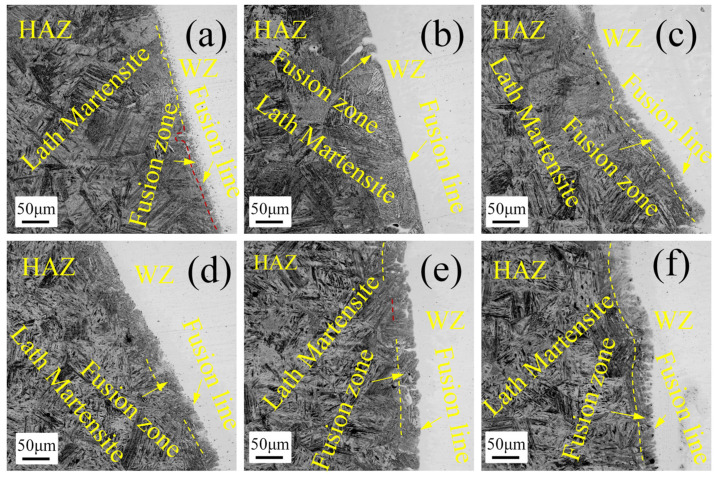
HAZ microstructure: (**a**–**f**) U0–U5.

The relevant studies have shown that the acoustic streaming effect generated by ultrasonic wave will affect the welding temperature and heat transfer and, finally, affect the HAZ near the weld [39]. Figure 8a–f correspond to the microstructures near the U0–U5 welds, respectively. In such a microstructure, there is a fusion zone where the coexistence of solid and liquid can be seen during the input of high welding heat. The microstructure is mainly composed of martensite-like layer and weld melt infiltration. A coarse-grained zone composed of coarse lath martensite is just adjacent to the fusion zone. As shown in Figure 8, with the increase of ultrasonic power, the width of the fusion zone becomes wider and the fusion line becomes bigger. This is because the ultrasonic wave will affect the welding energy. With the increase of ultrasonic power, the fluidity and diffusion capacity of weld melt are enhanced, and the infiltration of weld melt into the fusion zone becomes more serious [40]. At the same time, the ultrasonic streaming effect will improve the cooling rate of the weld. The ultrasonic vibration process itself will also generate a lot of heat, which can ensure a temperature environment in the process of weld cooling and result in the martensite refinement.

### 3.3. Microhardness Test

**Figure 9:**
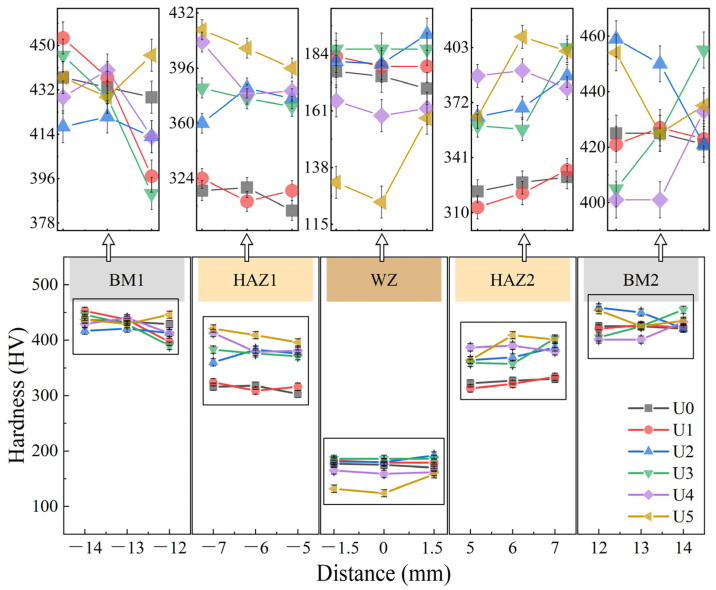
Microhardness distribution in a welded joint.

Through the above microstructure observation, it is found that ultrasonic power has a great influence on the microstructure of the weld and HAZ. This will inevitably affect the mechanical properties of a welded joint. The microhardness of BM, HAZ, and WZ zones was tested in six welded joint samples, namely U0 to U5. The test results are shown in Figure 9. The microhardness distribution in each sample is basically the same, i.e., the BM hardness is the highest, followed by HAZ hardness and WZ hardness. Specifically, the hardness values of different zones are quite different, which is directly related to the ultrasonic power. However, the influence of ultrasonic wave on the base metal is small. This study mainly discusses the microhardness of the weld and HAZ.

The HAZ microhardness increases with the ultrasonic power. The observation of HAZ1 and HAZ2 in Figure 9 indicates that the hardness values of the welds U0 and U1 are similar, while the hardness values of the U2–U5 welds increase in the order of U2–U5. This is caused by the mechanical properties of lath martensite. With the decrease of martensite size, the ability of phase interface to hinder the dislocation movement will increase, resulting in higher hardness. With the increase of ultrasonic power, the WZ microhardness will first increase and then decrease. U3 is the turning point of this state. The WZ microhardness values of the U4 and U5 welds decrease significantly below that of U0. According to the analysis of Figure 7 and Hall-Petch hardness relationship (Equation (1)), the reason for the hardness difference of the 6 groups of samples is that the ultrasonic wave has changed the size and distribution of grains, i.e., the hardness will increase with the decrease of grain size [41].

(1) σS=σ0+αSdg−12,

where *σ_S_* is the yield strength; *σ*_0_ is the frictional stress determined by crystal structure and dislocation density; *d_g_* is the average grain size; *α_S_* is the influence coefficient of grain boundary on strength; and *α_S_* is a constant.

### 3.4. Tensile Test

**Figure 10:**
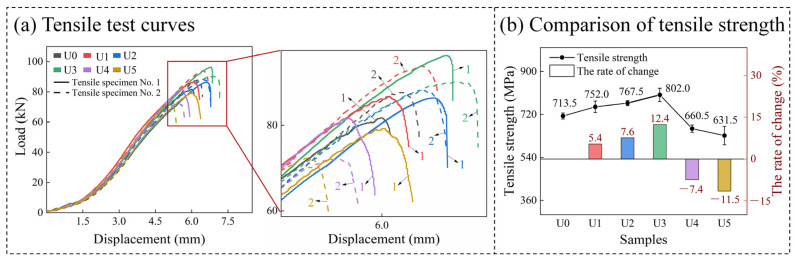
Tensile curves and tensile strength of the samples in different states.

In the tensile test of the samples, the strength of BM and HAZ is much higher than that of WZ, so the fracture position of all the tensile samples is WZ. Figure 10a shows the real tensile curves of 12 tensile samples in 6 groups. All curves have an inflection point at 1.5 mm displacement, and then rise sharply after the inflection point. When the ultrasonic power is U1 to U3, the slope of a curve will increase. With the increase of ultrasonic power, the tensile strength of the samples will also increase. The tensile strength of the two U1 specimens is basically the same, but the tensile strength of the No. 1 sample at U2 and U3 is stronger than that of the No. 2 sample. However, when the ultrasonic power is U4 and U5, not only the slope of the curves but also the tensile capacity of the samples will be reduced. The tensile capacity of the samples will decrease with the increase of ultrasonic power. The two tensile samples at U4 and U5 show the same trend as those at U1–U3, i.e., the tensile capacity of the No. 1 sample is stronger than that of the No. 2 sample. Figure 10b shows the change of average tensile strength of all samples relative to U0 tensile strength. The tensile strength of the U3 weld is the highest, up 12.4% from U0. The tensile strength of the U4 and U5 welds is greatly reduced. In particular, the tensile strength of the U5 weld is 11.5% lower than that of U0. This is because the acoustic steaming and cavitation effects generated by ultrasonic wave have regulated the grain state and macroscopic morphology of the welded joints [42].

According to the analysis of the sampling locations and exciter distribution in Figure 4a, the tensile property of a weld will be affected by not only ultrasonic power but also the position of ultrasonic transducer. It can be seen from the above results that the No. 1 tensile sample which is farther away from the transducer has higher strength than the No. 2 sample. This is because the No. 2 sample is located in the direct action zone of the ultrasonic transducer. When the ultrasonic power is low, the zone is less affected and is basically equivalent to the position of the No. 1 sample. However, with the increase of ultrasonic power, various complex effects generated by ultrasonic wave will become stronger in this zone, resulting in the performance difference between No. 1 and No. 2 tensile samples.

Theoretically, with the increase of ultrasonic power and transducers, the heat generated by welding can leave the base metal more easily and quickly to accelerate the solidification of the weld pool. This is the transfer of ultrasonic energy in the medium. On the other hand, the ultrasonic propagation enhances the fluidity of the melt, makes the temperature in the weld pool more uniform, slows down the temperature gradient for weld solidification, thus reducing the generated thermal residual stress and improves the tensile strength of the samples.

In this study, the average energy transmitted by ultrasonic transducer per unit area can be expressed as [27]:

(2) $$P=1/2\left(\lambda +2\mu \right)\frac{\omega^{2}}{V}A^{2},$$

where *λ* and *μ* are Lame constants, *V* is the velocity of ultrasonic propagation, and *A* is ultrasonic amplitude.

**Figure 11:**
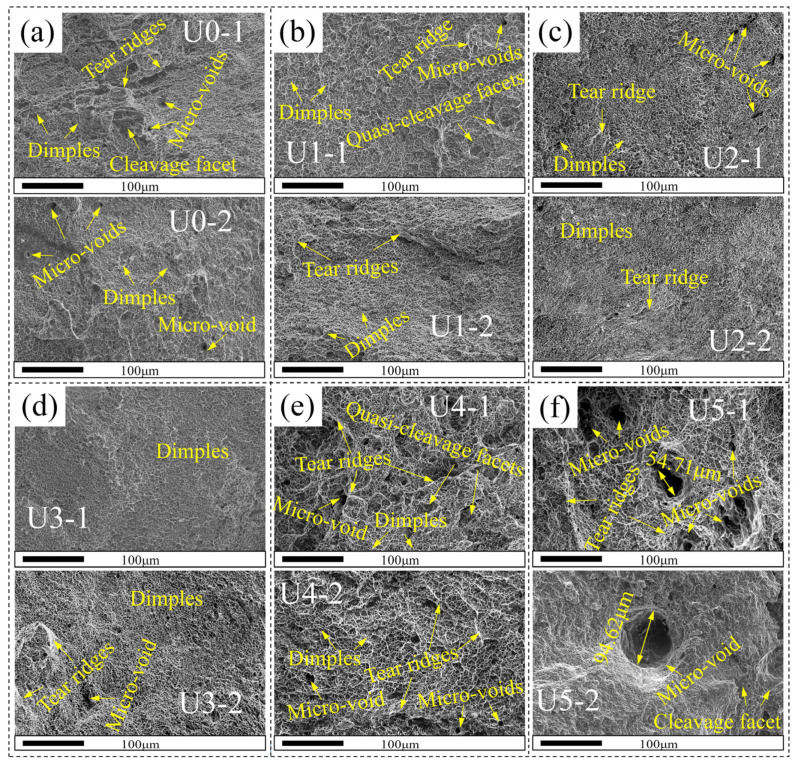
SEM picture of the fracture of a tensile sample: (**a**–**f**) U0–U5, respectively; 1 and 2 correspond to tensile specimen No. 1 and tensile specimen No. 2, respectively.

In order to further analyze the fracture mode and microscopic morphology of each tensile sample, the fracture of the welded joints was observed by SEM. The results are shown in Figure 11. The fracture morphology of all samples is dominated by dimples, which indicates that the fracture of all samples is ductile fracture. Partial cleavage planes and long tearing edges are seen in the fracture of the No. 1 U0 sample. In the U1, U2, and U3 welds, the dimples are finer and finer, and the tearing edges are shorter and shorter in the order of U1, U2, and U3. Among them, the dimples of the No. 1 U3 sample are the smallest and most uniform, showing the best mechanical properties that are consistent with the tensile test results. This mainly results from the refinement of columnar crystals and the uniform distribution of grains, which contributes to the anisotropy reduction. In addition, the uniform distribution of interdendritic ferrite is also conducive to preventing the propagation of microcracks in the tension process [43]. However, larger dimples and quasi-cleavage planes can be seen in the fractures of the U4 and U5 welds. Unfortunately, the fractures of all ultrasonically controlled welds have microdefects. When the ultrasonic power exceeds U3, the ultrasonic wave will cause a large area of defects. The microdefects in the No. 2 U5 sample are as large as 94.62 μm. On the other hand, this indicates that the ultrasonic wave may cause defects in the process of melt solidification. When the defects reach a certain size, the cracking can be formed easily under tensile stress, resulting in the reduction of tensile strength. This is quite different from the existing research results, possibly because of the material difference.

### 3.5. Analysis of Weld Element Content

**Figure 12:**
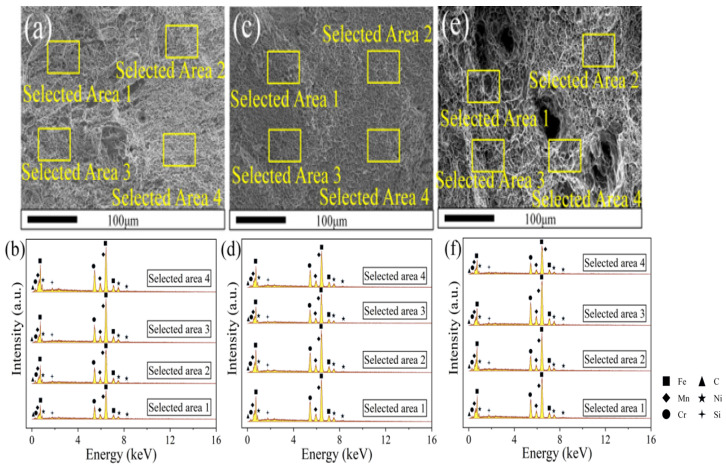
EDS energy spectrum analysis: (**a**,**c**,**e**) the fractures and EDS zones of the No. 1 sample at U0, U3, and U5, respectively; (**b**,**d**,**f**) the EDS energy spectra of the U0, U3, and U5 welds, respectively.

**Figure 13:**
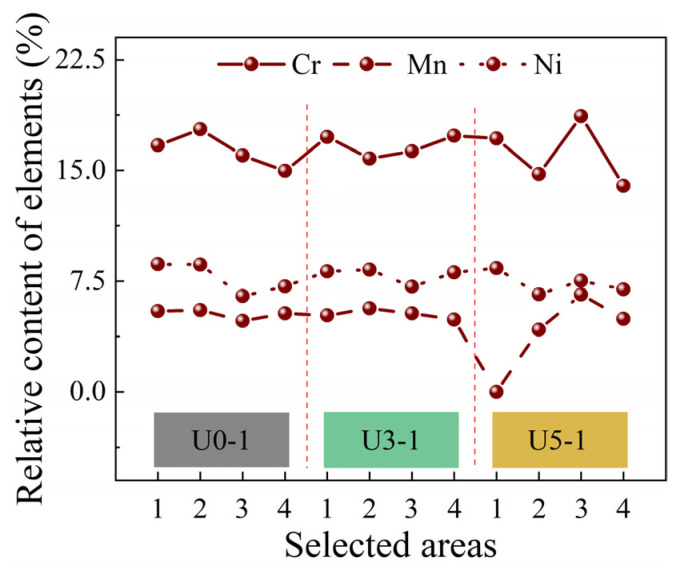
Effect of ultrasonic wave on element segregation.

The 6211 armor steel and stainless-steel welding wire have different microstructures and chemical compositions. During welding, the weld metal will be diluted. In addition, before the solidification of weld pool, the metal solution will flow with the driving force in the pool to cause the metal element segregation. The elements of stainless steel welding wire, such as Cr, Mn, and Ni, play a key role in deciding the mechanical properties of the weld. Therefore, the four zones on the fracture surface of the No. 1 tensile sample at U0, U3, and U5 are selected for element content analysis. The analysis results are shown in Figure 12 and Figure 13. The element segregation in the U0 weld is moderate, the element distribution in the U3 weld is uniform, and the element segregation in the U5 weld is serious. It can be seen that ultrasonic wave can regulate the element segregation but not very effectively. Moreover, when the ultrasonic power is very high, serious element segregation will be caused. This is because the ultrasonic waves have changed the melt surface tension and increased the melt flowability. When the ultrasonic power is higher, the melt in the weld pool will flow better and can be more easily and evenly distributed. However, a stream of very powerful ultrasonic waves can also quickly gather metal elements to cause serious segregation.

### 3.6. Impact Test

**Figure 14:**
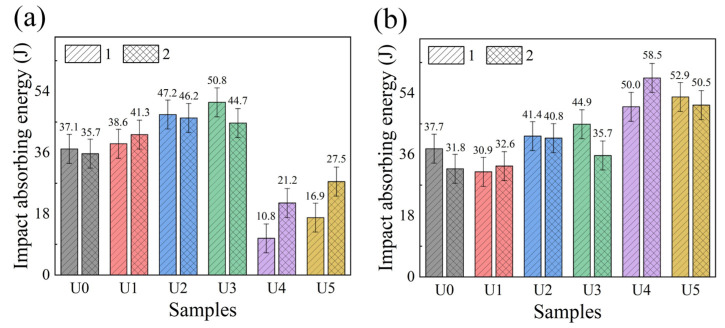
Impact absorption energy of different welded joints: (**a**) impact absorption energy of WZ; (**b**) impact absorption energy of HAZ.

Homogeneous armor steel is mainly used for weapons, and its impact resistance is very important for the overall product performance. Generally speaking, the WZ and HAZ of a 6211 welding structure have lower strength and can be damaged easily by an impact. In view of this situation, four impact samples were selected at the welded joint, as shown in Figure 4, including the WZ impact samples 1 and 2, and the HAZ impact samples 1 and 2. The test results are shown in Figure 14. The average impact absorption energy of WZ and HAZ in the U0 state is 36.4 J and 34.75 J, respectively. After the ultrasonic regulation at appropriate power, the WZ impact absorption energy of welded joints will increase gradually with the increase of ultrasonic power. The average WZ impact absorption energy in the U3 state is 47.75 J, which is significantly higher than that in the U0 state. However, high-power ultrasonic wave will cause a sharp decrease in the impact resistance of WZ, which is similar to the law of microhardness change. The impact resistance of HAZ increases with the ultrasonic power, but the maximum impact absorption energy appears in the U4 state. Therefore, ultrasonic power is not the only factor affecting the impact resistance of HAZ during the experiment.

## 4. Conclusions

In this study, the weld pool was regulated by applying the ultrasonic waves with different power in the welding process of 6211 homogeneous armor steel, and then the effect of ultrasonic power on the welded joint was analyzed by measuring the feature sizes in each zone of the joint and analyzing the microstructure, microhardness, tensile strength, impact strength, and other features of the joint. The detailed conclusions about these features are as follows:

(1) Ultrasonic wave has a significant influence on the surface-forming quality of a weld and the macroscopic morphology of the joint. With the increase of ultrasonic power, the weld pool spattering and the weld-forming quality will deteriorate. However, the use of ultrasonic waves can help increase the pool depth and can even help increase the pool width if the ultrasonic power is appropriate. Unfortunately, ultrasonic regulation will increase the HAZ width. The pool widths and HAZ widths are highly symmetric on both sides of an ultrasonic weld.
(2) It is beneficial to improve the microstructure of welded joints by using the ultrasonic waves with appropriate power for weld regulation during welding. The ultrasonic regulation with appropriate power cannot only refine the grains in WZ and HAZ and distribute the WZ grains evenly but also regulate the segregation of weld metal elements—but with a weak effect. High ultrasonic power will make the microstructure worse.
(3) In the six groups of welding experiments, the U3 weld zone has the best microhardness and impact resistance, as well as the highest tensile strength (802 MPa). The mechanical properties of the U1 and U2 weld zones are also improved to some extent, while those of the U4 and U5 weld zones are significantly degraded. However, the HAZs in the U4 and U5 states have the best microhardness and impact resistance. This also shows that different ultrasonic power can be used to regulate the welded joints with different performance requirements.

The study of the above features shows that the ultrasonic power used for weld pool regulation has a threshold. The ultrasonic power lower than the threshold will play a positive role in regulating the welded joint, while the ultrasonic power higher than the threshold will bring down the comprehensive performance of the joint.

## Figures and Tables

**Table 1:** Main chemical composition of base material and welding wire (% by mass).

Material	C	Si	Mn	P	S	Cr	Ni	Mo	Nb
Base material	0.27–0.31	0.20–0.35	0.20–0.35	≤0.015	≤0.008	0.60–1.00	0.60–0.85	0.15–0.30	0.02–0.06
Welding wire	0.04–0.14	0.65–1.00	6.50–8.00	≤0.030	≤0.030	18.50–22.00	8.00–10.75	≤0.75	—

**Table 2:** Welding parameters.

Parameters	Value
Base metal thickness (mm)	5
Welding current (A)	200
Welding voltage (V)	22
Welding speed (mm/s)	5
Gas flow (L/min)	15
Gas ratio	95% Ar + 5% CO_2_
wire extension (mm)	15

## Data Availability

Data are contained within the article.
